# Redescription of *Marstonia comalensis* (Pilsbry & Ferriss, 1906), a poorly known and possibly threatened freshwater gastropod from the Edwards Plateau region (Texas)

**DOI:** 10.3897/zookeys.77.935

**Published:** 2011-01-26

**Authors:** Robert Hershler, Hsiu-Ping Liu

**Affiliations:** 1 Department of Invertebrate Zoology, Smithsonian Institution, P.O. Box 37012, Washington, D.C. 20013-7012, USA; 2 Department of Biology, Metropolitan State College of Denver, Denver, CO 80217, USA

**Keywords:** *Marstonia*, Hydrobiidae, Gastropoda, United States, Texas, freshwater, taxonomy, conservation

## Abstract

Marstonia comalensis, a poorly known nymphophiline gastropod (originally described from Comal Creek, Texas) that has often been confused with Cincinnatia integra, is re-described and the generic placement of this species, which was recently allocated to Marstonia based on unpublished evidence, is confirmed by anatomical study. Marstonia comalensis is a large congener having an ovate-conic, openly umbilicate shell and penis having a short filament and oblique, squarish lobe bearing a narrow gland along its distal edge. It is well differentiated morphologically from congeners having similar shells and penes and is also genetically divergent relative to those congeners that have been sequenced (mtCOI divergence 3.0–8.5%). A Bayesian analysis of a small COI dataset resolved Marstonia comalensis in a poorly supported sub-clade together with Marstonia hershleri, Marstonia lustrica and Marstonia pachyta. The predominantly new records presented herein indicate that Marstonia comalensis was historically distributed in the upper portions of the Brazos, Colorado, Guadalupe and Nueces River basins, south-central Texas. The species has been live collected at only 12 localities and only two of these have been re-visited since 1993. These data suggest that the conservation status of this snail, which has a critically imperiled (G1) NatureServe ranking and was recently proposed for federal listing, needs to be re-assessed.

## Introduction

The freshwater gastropod genus Marstonia (Hydrobiidae: Nymphophilinae) is composed of 15 small (shell height < 5.0 mm), ovate- to elongate-shelled species that are distributed in springs, streams and lakes in eastern North America (Thompson and Hershler 2002, Hershler et al. 2003a, Thompson 2005). Most of these species have extremely narrow geographic ranges and consequently have become a focus of conservation activities; two are federally listed as endangered (USFWS 1994, 2000) and others are variously listed by state wildlife agencies. Marstonia differs from the other eight North American nymphophiline genera in that the (female) oviduct and bursal duct join well in front of (instead of behind) the posterior wall of the pallial cavity (Thompson and Hershler 2002); it has also been resolved as a well supported sub-clade within its subfamily based on mtDNA sequences (Hershler et al. 2003a). Although Marstonia has been reviewed three times since 1978 (Thompson 1978, Hershler 1994, Thompson and Hershler 2002), three of its congeners have been little studied beyond their original descriptions and their anatomy is unknown. Two of these — Marstonia olivacea (Pilsbry), Marstonia ozarkensis (Hinkley) — may be extinct (Thompson 1978, NatureServe 2009) and thus will likely remain *incertae sedis*. The third, Marstonia comalensis (Pilsbry & Ferriss) (originally Amnicola comalensis Pilsbry & Ferriss), which is extant, is the focus of this paper.

Pilsbry and Ferriss (1906) described Amnicola comalensis based on six shells from Comal Creek and the Guadalupe River near New Braunfels, south-central Texas. They differentiated this species from Amnicola limosa (Say) and two *nomina* (Amnicola cincinnatiensis [Anthony], Amnicola peracuta Pilsbry & Walker) that are currently recognized as synonyms of Cincinnatia integra (Say) (see Hershler and Thompson 1996) by its much smaller size and noted that it further differed from the latter by its less shouldered whorls. The genus Amnicola was used at that time as “a catch-all for most American amnicoloid species that could not conveniently be placed elsewhere on the basis of their shells” (Thompson 1968: 150). Amnicola comalensis was not further treated taxonomically until Taylor (1975) transferred it to Cincinnatia without comment in a bibliographic compilation; this allocation was widely followed in the subsequent literature (e.g., Burch and Tottenham 1980, Turgeon et al. 1998). During the course of a revisionary study of Cincinnatia integra, Hershler and Thompson (1996) examined several alcohol preserved collections of a snail that they identified as Amnicola comalensis and noted that it closely resembled species of Marstonia (which were then placed in Pyrgulopsis); Amnicola comalensis was subsequently transferred to Marstonia based on this unpublished work (Thompson and Hershler 2002). Hershler et al. (2003a) recently published a molecular phylogenetic analysis of the North American nymphophilines that included a specimen of Marstonia comalensis from Old Faithful Spring in Real County, Texas (ca. 180 km from the type locality), which was depicted as nested within the Marstonia clade. This is the only published record for Marstonia comalensis subsequent to its original description.

We redescribe Marstonia comalensis herein based on study of a large series of dry shell and alcohol-preserved material, most of which was collected by malacologists J.J. Landye and D.W. Taylor from 1971–1993, and provide anatomical evidence supporting its current generic allocation. The new records detailed in this paper considerably expand the geographic range of Marstonia comalensis, which lives in springs and fluvial habitats spread among four river basins in south-central Texas. We also further analyze previously published molecular data (Hershler et al. 2003a) to evaluate the divergence and phylogenetic relationships of Marstonia comalensis, whose geographic range is broadly disjunct relative to other members of the genus. The information presented in this paper may assist efforts to protect this poorly known species, which was included in a recent federal listing petition (Rosmarino and Tutchton 2007) based on its critically imperiled (G1) NatureServe (2009) ranking, but found not to warrant listing owing to insufficient information (USFWS 2009).

## Materials and methods

Anatomical study was based on specimens that were relaxed with menthol crystals and fixed in dilute formalin. Types and other material of Marstonia comalensis in the collections of the Academy of Natural Sciences of Philadelphia (ANSP); Florida Museum of Natural History (FMNH); National Museum of Natural History, Smithsonian Institution (USNM); and University of Minnesota Bell Museum of Natural History (UMBMNH) were examined during the course of this study.

Variation in the number of cusps on the radular teeth was assessed using the method of Hershler et al. (2007). Other methods of morphological study and descriptive terminology are those used in recent taxonomic investigations of nymphophiline gastropods (Hershler 1998, Hershler et al. 2003b). Shell data were compiled using Systat for Windows 11.00.01 (SSI 2004).

The molecular phylogenetic analysis included single mtCOI sequences from Marstonia comalensis, six other species of Marstonia and representatives of six other North American nymphophiline genera. Hydrobia acuta (Draparnaud) was used as the root. Sample information, GenBank accession numbers and publication references for the sequences are in Table 1. Sequence divergences (uncorrected p distance) were calculated using MEGA4 (Tamura et al. 2007). Phylogenetic relationships were inferred using Bayesian inference in MrBayes 3.12 (Ronquist and Huelsenbeck 2003). MrModeltest (Nylander 2004) selected the Hasegawa-Kishino-Yano model with some sites assumed to be invariable and with variable sites assumed to follow a discrete gamma distribution (HKY + I + G), which best fit the data under the Akaike Information Criterion. In the initial Bayesian analysis the burn-in was set at 10% (10,000 generations) of the chain length (100,000 generations). Three runs were conducted in MrBayes using the HKY + I + G model and the default random tree option to determine when the log-likelihood sum reached a stable value (by plotting the log-likelihood scores of sample points against generation time). The ln likelihoods started around -4,300 and quickly converged upon a stable value of about -3,050 after 1,000 generations. For the final run, Metropolis-coupled Markov chain Monte Carlo simulations were performed with four chains for 1,000,000 generations and Markov chains were sampled at intervals of 10 generations to obtain 100,000 sample points. The sampled trees with branch lengths were used to generate a 50% majority rule consensus tree with the first 5000 trees (equal to 50,000 generations) removed to ensure that the chain sampled a stationary portion.

**Table 1.** Species (specimen codes), locality details, GenBank accession numbers and publication references for mtCOI sequences.

*Species (code)*	*Locality*	*GenBank accession number*	*Reference*
Marstonia agarhecta Thompson	Bluff Creek, Pulaski Co., GA	AF520934	Hershler et al. 2003a
Marstonia castor Thompson	Mercer Mill Creek, Worth Co., GA	AF520938	Hershler et al. 2003a
Marstonia comalensis (Pilsbry & Ferriss)	Old Faithful Spring, Real Co., TX	AF520933	Hershler et al. 2003a
Marstonia halcyon Thompson	Ogeechee River, Screven Co., GA	AF520935	Hershler et al. 2003a
Marstonia hershleri (Thompson)	Coosa River, Elmore Co., AL	AF520946	Hershler et al. 2003a
Marstonia lustrica (Pilsbry)	Stockbridge Bowl, Berkshire Co., MA	AF520945	Hershler et al. 2003a
Marstonia pachyta Thompson	Limestone Creek, Limestone Co., AL	AF520939	Hershler et al. 2003a
Cincinnatia integra (Say)	Stream north of Fredericksburg, Gillespie Co., TX	AF520948	Hershler et al. 2003a
Notogillia wetherby (Dall) (WW)	Weeki Wachee River, Hernando Co., FL	AF367630	Wilke et al. 2001
Notogillia wetherbyi (Dall) (RS)	Rainbow Springs, Marion Co., FL	AF520918	Hershler et al. 2003a
Pyrgulopsis bruneauensis Hershler	Bruneau Hot Springs, Owyhee Co., ID	AF520941	Hershler et al. 2003a
Rhapinema dacryon Thompson	Chipola River, Jackson Co., FL	AF520932	Hershler et al. 2003a
Spilochlamys gravis Thompson	Alexander Springs, Lake Co., FL	AF520919	Hershler et al. 2003a
Stiobia nana Thompson	Coldwater Spring, Calhoun Co., AL	AF520921	Hershler et al. 2003a
Hydrobia acuta (Draparnaud)	Lagoon 6, Suffolk, East Anglia, United Kingdom	AF354773	Liu et al. 2001

Additional locality details are in Hershler et al. (2003a).

## Systematic description

**Family Hydrobiidae**

**Subfamily Nymphophilinae**

**Genus Marstonia Baker, 1926**

### Marstonia comalensis

(Pilsbry & Ferriss, 1906)

Figs 1 [Fig F2] 3

Amnicola comalensis  Pilsbry & Ferriss, 1906: 171, fig. 37 (Comal Creek, near New Braunfels, Comal County, Texas; also from the Guadalupe River about four miles [3.2 km] above New Braunfels). Pilsbry 1910: 98 (corrected measurement of figured specimen [lectotype]). Walker 1918: 133. Baker 1964: 172 (lectotype selection). Hershler and Thompson 1996: 51.
Cincinnatia comalensis Taylor 1975: 61 (transfer to Cincinnatia, summary of literature citations). Burch and Tottenham 1980: 110, fig. 190 (from Pilsbry and Ferris 1906). Turgeon et al. 1998: 72.
Marstonia comalensis Thompson and Hershler 2002: 270 (transfer to Marstonia). Hershler et al. 2003a: 366, figs. 2, 3 (new record, phylogenetic analysis).

#### Types:

**Figure 1.**
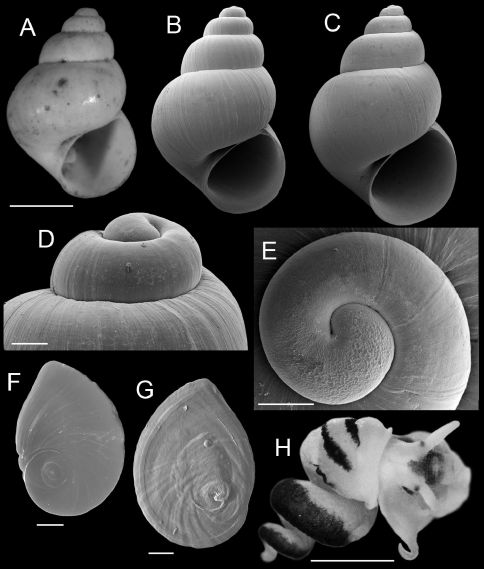
Shells, opercula and animal, Marstonia comalensis. **A** Lectotype, ANSP 91323 **B, C** Shells, USNM 874932, USNM 874926 **D** Lateral view of shell apex, USNM 874932 **E** Protoconch, USNM 874926 **F, G** Opercula (outer, inner sides), USNM 874926 **H** Relaxed, alcohol-preserved preserved male (dorsal view), showing distinctive pigment bands on the pallial roof, USNM 874926. Scale bars A–C, H, 1.0 mm; D, E, 100 µm; F, G, 200 µm.

Figured lectotype, ANSP 91323 (Fig. 1A); paralectotypes (from same lot), ANSP 420575.

#### Referred material:

TEXAS. USNM 123757, USNM 134007, Guadalupe River. ANSP 134247, Nueces River. *Bell County*: UMBMNH uncat., Salado Creek, Salado, old US 81 (30.944°N, 97.539°W), 14.IV.1972. *Comal County*: ANSP 90562, drift of Guadalupe River, 3.2 km above New Braunfels (29.756°N, 98.138°W). UMBMNH uncat., Spring Branch, west of Spring Branch (29.891°N, 98.435°W), 28.III.1963. USNM 473488, New Braunfels (29.702°N, 98.124°W). *Kerr County*: USNM 874910, South Fork Guadalupe River, ca. 56.4 km northwest of Leakey (29.957°N, 99.456°W), 30.XII.1979. USNM 874932, spring run adjacent to South Fork of Guadalupe River, ca. 11.2 km southwest of Hunt (30.005°N, 99.409°W), 30.XII.1979. USNM 883412, North Fork Guadalupe River (30.054°N, 99.486°W), 26.IV.1993. USNM 874923, North Fork of Guadalupe River at Riverbend Ranch crossing at FM 1340 (30.065°N, 99.373°W), 29.XII.1979. USNM 251887, Japonica (30.064°N, 99.344°W). *Kimble County*: UMBMNH uncat., Llano River at FM 385, 25.6 km northeast of Junction (30.589°N, 99.598°W), 12–13.IV.1972. *Kinney County*: UMBMNH uncat., Nueces River, Tularosa Road, A.G. “Tony” Rose Ranch (29.518°N, 100.271°W), 9.VI.1971. USNM 883413, West Nueces River above crossing on Tularosa Road near Spring Ranch, just below Silver Lake (29.523°N, 100.251°W), 25.IV.1993. *Real County*: UMBMNH uncat., USNM 874926, Old Faithful Spring outflow, Hwy 55, 0.8 km north of Camp Wood (29.680°N, 100.015°W), 8.VI.1971, 31.XII.1979. FMNH 283564, FMNH 283565, FMNH 283573, FMNH 287574, Old Faithful Spring, 1.0 km north of Camp Wood (29.680°N, 100.015°W), 27.I.2001. USNM 883414, Leakey Springs run at Hwy 337 crossing, ca. 0.48 km east of Leakey (29.723°N, 99.757°W), 25.IV.1993. FMNH 283561, Leakey Springs creek, 0.64 km east of Leakey (29.723°N, 99.757°W), 26.I.2001. *Uvalde County*: UMBMNH uncat., Nueces River, Chalk Bluff, 24 km northwest of Uvalde (29.359°N, 99.984°W), 27.V.1971. USNM 883477, spring at Camp Chalk Bluff, tributary to East Nueces River (29.362°N, 99.984°W), 23.IV.1993. USNM 883666, USNM 883421, East Nueces River at 19 Mile Crossing, 1.6–3.2 km south of Hwy 55 and FM 334 (29.398°N, 100.00°W), 31.XII.1979, 29.IV.1993. UMBMNH uncat., USNM 883420, East Nueces River, Hwy 55, Lake Nueces County Park (29.619°N, 100.01°W), 1-VI-1971, 29.IV.1993.

#### Revised diagnosis:

Shell large for genus (maximum height, 4.6 mm), ovate-conic, openly umbilicate; penis with short filament and oblique, squarish lobe bearing a single terminal gland along its distal edge. Distinguished from congeners having closely similar shells and penes as follows: from Marstonia gaddisorum Thompson by its less convex shell whorls, distinctive pallial roof pigmentation, larger number of cusps on the inner side of the lateral teeth and on the outer marginal teeth, larger penial lobe, narrower terminal gland, and smaller overlap of the bursa copulatrix by the albumen gland; from Marstonia lustrica by its smaller prostate gland, smaller penial lobe, narrower penial filament, straight anterior vas deferens, partly imbedded (in albumen gland) bursal duct, and larger seminal receptacle; and from Marstonia ogmorhaphe Thompson by its smaller size, broader shell, smaller prostate gland, straight anterior vas deferens, and smaller bursa copulatrix.

#### Description:

Shell ovate-conic, (Fig. 1A–C); height about 2.6–4.6 mm; whorls 4.5–5.5. Protoconch near planispiral, slightly tilted (Fig. 1D), initial 0.75–1.0 whorl strongly wrinkled (Fig. 1E). Teleoconch whorls weakly convex, often narrowly shouldered, rarely having subsutural angulation; sculpture of strong collabral growth lines, later whorls having numerous weak spiral striae. Aperture pyriform or ovate. Inner lip complete across parietal wall in larger specimens, usually narrowly adnate, rarely slightly disjunct; usually thin, sometimes slightly thickened apically; columellar shelf absent or very narrow; outer lip thin or slightly thickened, orthocline or prosocline. Umbilicus open but small. Measurements of the lectotype and a live-collected series of shells from the Nueces River basin are in Table 2.

**Figure 2. F2:**
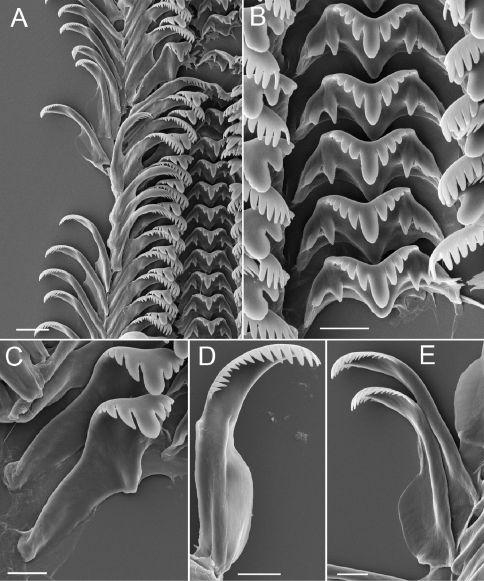
Radula, Marstonia comalensis, USNM 874926. **A** Portion of radular ribbon **B** Central teeth **C** Lateral teeth **D** Inner marginal tooth **E** Outer marginal teeth. Scale bars A, 20 µm; B-E, 10 µm.

Operculum thin, amber, narrowly ovate, multispiral with eccentric nucleus (Fig. 1F); last 0.25 whorl sometimes frilled on outer side; inner side having well developed rim near outer edge (Fig. 1G); attachment scar border sometimes weakly thickened near nucleus. Radula (Fig. 2A), having about 36 well-formed rows of teeth. Central teeth about 38 µm wide, cutting edge convex (Fig. 2B); lateral cusps 3–8; central cusp pointed or hoe-shaped, parallel-sided proximally or tapering throughout; basal cusps 1–3, small; basal tongue U- or V-shaped, about as long as lateral margins. Lateral tooth face rectangular; central cusp pointed or hoe-shaped (Fig. 2C); lateral cusps 2–5 (inner), 3–7 (outer); outer wing broad, flexed, about 140% length of cutting edge; basal tongue weakly developed. Inner (Fig. 2D) and outer (Fig 2E) marginal teeth both having 14–21 cusps and basally positioned rectangular wing. Radular count data were from USNM 874926.

**Figure 3.**
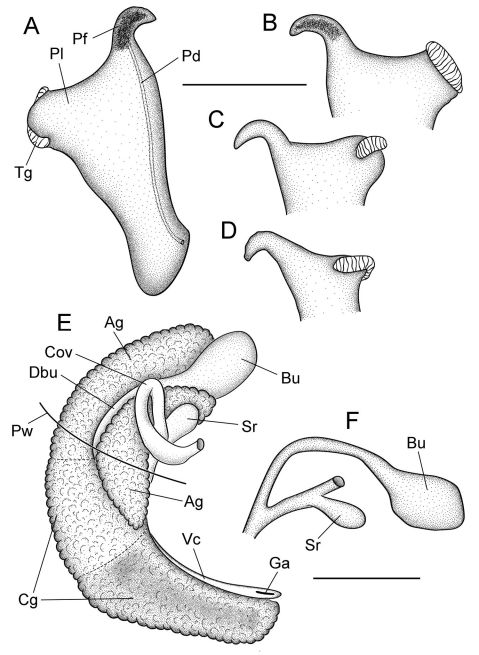
Reproductive anatomy, Marstonia comalensis, USNM 874926. **A** Penis, dorsal surface **B–D** Distal portion of penis showing terminal gland variation **E** Female glandular oviduct and associated structures (viewed from left side) **F** Bursa copulatrix and seminal receptacle and their ducts. Scale bars = 500 µm. **Ag** albumen gland **Bu** bursa copulatrix **Cg** capsule gland **Cov** coiled oviduct **Dby** bursal duct **Ga** genital aperture **Pf** penial filament **Pl** penial lobe **Pw** posterior wall of pallial cavity **Sr** seminal receptacle **Vc** ventral channel of capsule gland.

Cephalic tentacles pale except for black eyespots. Snout brown; distal lips pale; foot pale. Pallial roof having black pigment bands along edges of ctenidium and dorsal edge of genital duct (Fig. 1H); visceral coil pale except for black pigment on testis. Ctenidium positioned a little in front of pericardium; ctenidial filaments 24–25 (*n*=5), broadly triangular, lateral surfaces ridged. Osphradium narrow, positioned slightly posterior to middle of ctenidium. Hypobranchial gland large, overlapping rectum and part of genital duct, thickened alongside kidney. Style sac longer than remaining portion of stomach, posterior stomach having small caecal appendix. Testis large (1.75 whorls), composed of compound lobes, broadly overlapping stomach anteriorly. Seminal vesicle opening near anterior edge of testis, composed of a few thickened coils, positioned along ventral side of anterior 33% of testis. Prostate gland small, pea-shaped, with about 50% of length in pallial roof. Anterior vas deferens opening from antero-ventral edge of prostate gland, section of duct on columellar muscle straight. Penis large, base rectangular, inner edge without folds; filament short, narrow, tapering, oblique; lobe rather medium-sized, squarish, oblique (Fig. 3A). Terminal gland (Fig. 3A–D) narrow, usually transversely positioned along outer edge of lobe (58/86 specimens examined from three samples), less frequently horizontal (28/86), sometimes borne on short stalk. Penial duct narrow, near outer edge, almost straight. Penial filament having black internal pigment core along most of length. Ovary small (0.75 whorl), composed of simple, stalked lobes; slightly overlapping stomach anteriorly. Female glandular oviduct and associated structures shown in Figure 3E–F. Coiled oviduct narrow, vertical. Bursa copulatrix small, ovate, horizontal, about 50% overlapped by albumen gland. Bursal duct longer than bursa, narrow, opening from distal edge, partly embedded in albumen gland proximally, entirely embedded distally, junction with common duct well in front of posterior wall of pallial cavity. Seminal receptacle small, pouch-like, positioned near ventral edge of albumen gland slightly anterior to bursa copulatrix. Albumen gland largely visceral. Capsule gland composed of two distinct tissue sections. Genital aperture a terminal slit.

**Figure 4.**
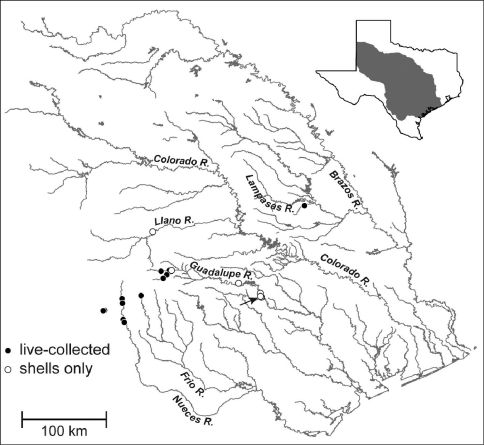
Map showing the distribution of Marstonia comalensis in the Brazos, Colorado, Guadalupe and Nueces River basins, south-central Texas. The arrow indicates the type locality (Comal Creek).

#### Distribution and habitat:

Marstonia comalensis is distributed in the upper portions of the Brazos, Colorado, Guadalupe and Nueces River basins, south-central Texas (Fig. 4); almost all of these localities are on the Edwards Plateau. We were unable to confirm a previous report of this species from a drainage canal near Galveston Bay (Cable and Isserhoff 1969). Marstonia comalensis lives in cold water springs near their sources and slack water riverine habitats; it has been most commonly found on mud, aquatic vegetation and dead leaves.

**Table 2.** Shell parameters for Marstonia comalensis.

	*WH*	*SH*	*SW*	*HBW*	*WBW*	*AH*	*AW*	*SW/SH*	*HBW/SH*	*AH/SH*
Lectotype, ANSP 91323										
	4.5	3.00	2.08	2.12	1.81	1.36	1.14	0.69	0.71	0.45
USNM 874926 (n=30)										
Mean	4.79	3.49	2.39	2.58	2.08	1.66	1.35	0.69	0.74	0.48
S.D.	0.18	0.17	0.09	0.11	0.08	0.09	0.05	0.03	0.02	0.02
Range	4.5–5.0	3.20–3.87	2.22–2.55	2.38–2.84	1.93–2.25	1.50–1.84	1.26–1.45	0.63–0.74	0.70–0.77	0.43–0.51

Abbreviations:

WH total shell whorls;

SH shell height;

SW shell width;

HBW height of body whorl;

WBW width of body whorl;

AH aperture height;

AW aperture width.

#### Remarks:

The material referred to Marstonia comalensis herein, which includes specimens from the Guadalupe River above the Comal Creek confluence, closely conforms to the types of this species both in size and shell shape (Fig. 1A–C). This snail clearly belongs to Marstonia based on its strongly wrinkled shell protoconch, distally bifurcate penis ornamented with a gland along the edge of the lobe (terminal gland), and connection between the oviduct and bursal duct well in front of the posterior pallial wall (Thompson and Hershler 2002). As noted above, this generic placement is also supported by molecular phylogenetic evidence (Hershler et al. 2003a).

The original collections of Marstonia comalensis are worn shells having the appearance of drift material (Fig. 1A). We have not seen any live-collected specimens of this species in the numerous samples that we have examined from the type locality (Comal Springs) and other waters near New Braunfels. The various reports of living Marstonia comalensis from this portion of the Guadalupe River basin (e.g., EHA 1975; Arsuffi 1994; Tolley-Jordan and Owen 2008) are probably of misidentified Cincinnatia integra, as evidenced by the illustrations of shells in several of these documents (Lindholm 1979, fig. 4; Cauble 1998, fig. 7). It is possible that Marstonia comalensis became extinct at Comal Springs when this water body temporarily dried in 1964 (USFWS 1996); it is also possible that the shells of this species which have been found at this site were washed downflow from extant populations in the headwaters of the Guadalupe River.

Taylor’s (1975) allocation of Amnicola comalensis to Cincinnatia appears to have been the result of a misidentification as all of the material in his collection that he referred to this species (per the original labels), including several lots from the type locality, is Cincinnatia integra (RH unpublished). Some of these records were detailed in an unpublished manuscript, “Freshwater molluscs from the Nueces River drainage, Texas” that Taylor circulated in the mid-1970’s. Cincinnatia integra, which is widely distributed in Texas (Hershler and Thompson 1996), has been frequently confused with Marstonia comalensis in museum collections despite the obvious differences between their shells that were noted in the original description of the latter (Pilsbry and Ferriss 1906). These two species also well differentiated anatomically (see Hershler and Thompson 1996 for details of the former).

## Discussion

**Figure 5.**
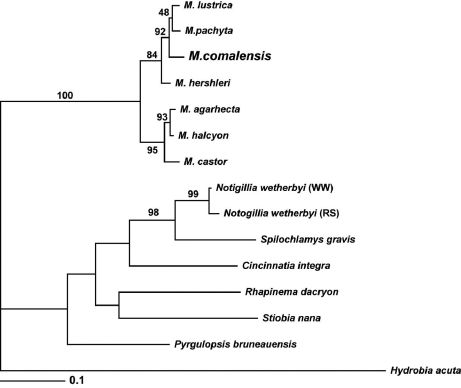
Bayesian tree based on the COI dataset. Posterior probabilities for each node within the Marstonia clade are shown for the reader’s interest; only those >95% are considered significant.

Thompson (1978) speculated that Marstonia is composed of two species lineages that are differentiated by the size and shape of the penial lobe and filament. Marstonia comalensis, which is distributed almost 800 km distant from its most proximal congener, conforms to the putative lineage characterized by a large, squarish lobe and small, slender filament. This group includes widely ranging Marstonia lustrica and species distributed in the Tennessee (Marstonia arga Thompson, Marstonia ogmorhaphe, Marstonia pachyta), Alabama (Marstonia hershleri; see Thompson 1995) and Altamaha (Marstonia gaddisorum; see Thompson 2005) River basins. The molecular phylogenetic relationships of Marstonia comalensis were previously delineated by Hershler et al. (2003a), who analyzed a mtCOI dataset using maximum parsimony and maximum likelihood methods. We re-analyzed the relevant portion of these data using a Bayesian algorithm The resulting topology (Fig. 5) is closely similar to those illustrated by Hershler et al. (2003a: figs. 2, 3) and has slightly better resolution. Marstonia is resolved as a well supported (100% posterior probability) clade. Three congeners living in Georgia coastal drainage, which Thompson (1978) recognized as a distinct lineage based on their elongate penial lobe and robust filament, formed a well supported (95%) sub-clade. The other four congeners included in the analysis, which conform to the second putative lineage discussed above, formed a weakly supported subunit, within which Marstonia comalensis was positioned as sister to a subclade containing Marstonia lustrica and Marstonia pachyta. The single sequenced specimen of Marstonia comalensis differs from those of the six other congeners that have been similarly analyzed by 3.0–8.5%; it is most similar to Marstonia pachyta.

Marstonia comalensis is ranked as critically imperiled (G1) by NatureServe (2009), but has a minimal conservation profile otherwise. Following the negative finding per its proposed listing (USFWS 2009) it was categorized by the USFWS (2010) as “status undefined.” It was misidentified as a “mussel” in a ecological sustainability report for the Cibola National Forest Grasslands plan revision (USDAFS 2008) and is not mentioned on the Texas Parks and Wildlife’s website (http://www.tpwd.state.tx.us/) or in the State Wildlife Action Plan (Texas Parks and Wildlife 2010). Given that Marstonia comalensis has been live-collected at only 12 localities (Fig. 4) and only two of these sites (Leakey Springs, Old Faithful Spring) have been re-visited since 1993, it would seem prudent to add this species to the list of aquatic biota of the Edwards Plateau region meriting protection (Bowles and Arsuffi 1993) and assess its current conservation status. Depending on the extent of its possible decline since 1993, Marstonia comalensis may merit addition to the IUCN Red List (e.g., if it consists of 10 or fewer extant populations; IUCN 2001) and other conservation watch lists.

## Supplementary Material

XML Treatment for Marstonia comalensis
